# Trusting social robots

**DOI:** 10.1007/s43681-022-00165-5

**Published:** 2022-05-24

**Authors:** Paula Sweeney

**Affiliations:** University of Aberdeen, Aberdeen, Scotland

**Keywords:** Artificial agents, Social robots, Trust, Fictional dualism, Reliability

## Abstract

In this paper, I argue that we need a more robust account of our ability and willingness to trust social robots. I motivate my argument by demonstrating that existing accounts of trust and of trusting social robots are inadequate. I identify that it is the feature of a façade or deception inherent in our engagement with social robots that both facilitates, and is in danger of undermining, trust. Finally, I utilise the fictional dualism model of social robots to clarify that trust in social robots, unlike trust in humans, must rely on an independent judgement of product reliability.

## Introduction

In this paper, I argue that we need a more robust account of our ability and willingness to trust social robots. We might think that we can simply extend existing theories of trust to accommodate trusting social robots. However, trust is generally considered to be a deeply anthropomorphic attitude that cannot be bestowed upon objects. As social robots are objects we would expect to be unable to take an attitude of trust towards them forming only a judgement of reliability, yet there is evidence that strongly suggests that we can and do take attitudes of trust towards social robots and this ability greatly enhances their usefulness in society.

The facilitator of the trust we bestow on social robots appears to be their ability to mimic human-to-human social behaviour. However, it is an expected link between human behaviour (the outer) and human attitudes (the inner) that facilitates trust between humans and, as this link is missing in the case of social robots, our willingness to trust them might be undermined by what amounts to a façade of agency. What we need, and what I provide, is some way to hold our attitude of trust accountable to both the robot’s social behaviour (the outer) and its functionality and design (the inner). In this way, we can temper our judgement of trust based on the appearances of social robots with a judgement of reliability regarding the robot as a product.

For the purpose of this paper, we can think of a social robot as a robot that interacts in what is interpreted as a social way and that can appear to present actions as being the result of intentions. The therapeutic baby seal, PARO (physically-assistive robots), is an example of a social robot. It was designed with social interaction in mind. It responds autonomously to being touched and to verbal triggers in a way that can reduce stress in elderly patients [13]. Other robots have been found to elicit a significant social response from humans despite such a response not being an intended feature of their design. The Roomba vacuum cleaner, for example, appears to take the place of a pet in some households, when it is given a name and engaged in (one-sided) conversation (Ja-Young et al.: 2007) Although Roomba was not designed with animal-like features and it does not communicate, its unpredictable and autonomous movements appear to lead to anthropomorphism. As such, a Roomba can also be considered to be a social robots.

I motivate my argument by demonstrating that existing accounts of trust and of trusting social robots are inadequate. I identify that it is the feature of a façade or deception inherent in our engagement with social robots that both facilitates and is in danger of undermining trust. Finally, I utilise the fictional dualism model of social robots to clarify that trust in social robots, unlike trust in humans, must rely on an independent judgement of product reliability. In Sect. 2, I highlight the anthropomorphic features of mainstream philosophical theories of trust and ask how these features might be compatible with our taking an attitude of trust towards social robots. In Sect. 3, I present some empirical evidence for the claim that humans are in fact capable of taking such an attitude towards social robots. In Sect. 4, I consider a proposal put forward by Mark Coeckelbergh, that trust is built on social relations and that we can have these relations towards social robots in virtue of their presenting to us as social beings. It is this presentation of social robots that makes our trust in them possible. Whilst I agree with Coeckelbergh’s proposal that we are capable of bestowing trust on social robots, I disagree that this capability is the end of the story with regards to the appropriateness of our trust in social robots. As Jonathan Tallant indicates, in order for trust to be appropriate, an appearance of agency that is based on deception is not enough. An expanded argument showing how the very facilitator of trust can also undermine it is given in Sect. 5. In Sect. 6, I build on the Fictional Dualism model of social robots [1] to show how trust in social robots may be both possible and appropriate. I further utilise the fictional dualism model in Sect. 7, reintroducing the notion of reliability and showing how an implicit assumption of reliability in the object as a product is essential for trust in the social presentation. This allows us to set appropriate limits or safeguards on the trust that we bestow. In Sect. 8, I summarise the consequences of my position regarding trusting social robots.

## Trust and anthropomorphic qualities

As philosophers worked to distinguish trust from reliance*,* philosophical views of trust became rooted in anthropomorphic features: whatever trust is, it must be distinguished from the attitude of reliance that we can have towards inanimate objects. We might rely on the ladder to hold us when cleaning out the gutters, but it would be incorrect to say that we trust the ladder.^1^

At the root of the distinction for many theorists is the claim that trust, unlike reliability, involves some kind of attitudinal state that both the truster and the trustee enter into. Holton, for example, believes that in trusting someone you rely on them and you regard that reliance in a certain way, with a readiness to feel betrayal should your reliance be disappointed ([15], p. 66) He states ‘In cases where we trust and are let down, we do not just feel disappointment, as we would if a machine let us down. We feel betrayed.’ For O’Neil ([17], p. 5), trust connects to gratitude. It involves an expectation that the trusted will discharge their commitment to us. It is through this expectation that the trusted are to feel honoured and grateful towards us. And it is the expected feeling of honour and gratitude from the trusted party that leads us to feel betrayed if they do not follow through. Bestowing trust is like giving a gift—one expects it to be gratefully received and valued.

Even for so-called ‘rationality’ approaches, such as the encapsulated interests account put forward by Russell Hardin [12], the trusted party plays an active role. When we might be required to trust someone, according to Hardin, we consider whether it is rational to expose ourselves to risk with this person and, in doing so, we form an expectation that they will encapsulate our interests into theirs because it will ultimately be of benefit to them to do so. I trust you because I know that you have my interests at heart to some extent. And you have my interest at heart because it is within your interest to do so—the inclination to cooperate with each other is reciprocal.

So trust, as it is generally understood by theorists, invokes an anthropomorphic form of commitment between the truster and the trustee. To return to Hawley’s example, I could not trust the ladder because it would make no sense for me to feel betrayed by the ladder if it failed under my weight as the ladder did not make a commitment to me. Likewise, it would make no sense for me to reason about the ladder encapsulating my interests within its own. Or, in Holton’s terms, I could not trust the ladder because it would make no sense for me to feel betrayed by the ladder, given that the ladder could not be reasonably expected to know that I am relying on it. The ladder could not meet the requirement for trust—it cannot be aware that I am relying on it, it cannot be reasonably expected to know that I am relying on it, it could not encapsulate my interests and could not feel honoured or gratified if I choose to rely on it.

In the attempt to differentiate trust from reliability, these conditions that theorists have introduced for trust are generally understood to exclude our trusting objects.^2^ Yet, it seems, we do trust social robots.^3^

## Trust in social robots

There is evidence to suggest that we do increasingly develop the kinds of relationships that are compatible with attitudes of trust towards social robots as they play a larger role supporting us in our lives: in healthcare, for entertainment and for personal support. The therapeutic healthcare robot PARO, introduced above, is an example of a social robot that is designed to provide healthcare support to elderly people, particularly those with dementia. Through its personalised interactions with the patient, PARO builds a relationship that develops and grows with increased familiarity and interactions [13]. During the COVID-19 lockdown period there were studies reporting that lonely people can gain comfort and companionship through interactions with robots. Two robot-based studies conducted by Christopher Williams et al. [22] concluded that interactions with robotic dogs significantly reduced loneliness and, in fact, that interactions with robotic and living dogs led to similar reductions in loneliness. De Graff, Allouch and Klamer [9] report on a significant long-term explorative study in which they look at the acceptance of social robots in domestic environments by older (50+) adults. The researchers are interested in understanding trust between humans and social robots to make relationship building easier. This is important because it is only if humans accept robots into their lives that they can reap the full potential of the technology. The study provides qualitative evidence of relationship building and trust between the human participants and their social robots. For example, when asked about their interactions, there is evidence that some participants treat the robot, which has the appearance of a large robotic rabbit, as more than an object:

‘I know I have said to him [the robot] on Saturday, ‘I have not much time to speak with you for the simple reason that [her son] is coming and I have got to give him my priority.’

And,

‘I must have said some funny things to the rabbit […], especially if I wasn’t sleeping very well and I’d come down in the middle of the night.’

Others noted how they missed the robot when the study ended:

‘We missed her [the robot]. Oh yes. […] She had been given a personality.’

And,

‘I missed him [the robot] for the first couple of days.’

And, more closely relating to trust:

‘I only trusted it [the robot] when it believed me.’

And,

‘I suppose, in the long term, I had accepted him [the robot] into my house.’

Through indirect evidence of the benefits that social robots can bring as a result of their close interactions with humans and through the more direct qualitative evidence given above, it seems that we can take an attitude of trust towards social robots.^4^ The tasks that social robots are being expected to undertake—care giving, prevention of loneliness, social engagement—might reasonably be considered to have trust as a prerequisite. As Taddeo puts it, ‘Trust is a facilitator of interactions among the members of a system.’ ([20]: 2), and there is much evidence to suggest that our social system now includes social robots. It is, I propose, plausible that as social robots appear to us to be more animal or human-like, the boundaries that existed that made trust appropriate for humans but not for objects are becoming blurred to the extent that they are under significant pressure. Arguably, we are granting trust to social robots without a good understanding of the basis of our attitude.

## Trust on the basis of appearances

In ‘Can we trust robots?’ (2012) [4], Coeckelbergh questions what trust can mean in relation to robots. Considering various traditional accounts of trust he notes that robots as entities do not possess the qualities of agency that these accounts standardly assume to be prerequisites for trust.^5^ However, he then highlights the fact that, when it comes to social robots, it may not really matter whether they can legitimately count as agents, but rather whether they appear to us as agents or, at least, as more than objects. He articulates a phenomenological-social approach to trust.

‘We trust robots if they appear trustworthy and they appear trustworthy if they are good players in the social game.’ (58)

For Coeckelbergh, it is their ability to participate in and shape our social dimension that sets the conditions for our relationships with social robots. To be eligible recipients of trust, and not just reliability, robots must fulfil criteria regarding *the appearance* of language use, freedom and social relations. Trust arises towards social robots because they appear to us to be human- or animal-like. Because of how they appear, we treat robots as if they were persons or pets, and this includes trusting them. Thinking back to the anthropomorphic features that we outlined above, we might say that trust does not require that some attitude has been taken by the trusted party, rather it requires the presentation of behaviour that is indicative of some attitude being taken by the trusted party.

So, for Coeckelbergh, what may facilitate trust in this area is what social robots appear to be, not what they, in fact, are. On the face of it, this seems plausible and would account for the evidence of trusting social robots that that we considered above. Because social robots present to us as having anthropomorphic features and, perhaps more importantly, because we respond to these presentations as if the object itself was really capable of forming attitudes, we are capable of forming an attitude of trust towards social robots.

However, having accepted that we are capable of forming attitudes of trust towards social robots the question then arises, should we? That is, is it not the case that we are mistakenly bestowing trust on objects in virtue of their appearing to be something that they are not? If so, does not that undermine the trust? In asking this we are not asking the important but different question of whether or not social robots are trustworthy—a value question—but rather asking whether social robots are the kinds of objects that it would be appropriate for us to form an attitude of trust towards, under the right conditions. Social robots are essentially presenting to us as something that they are not. The devices that are designed to be robotic companions—whether they are robotic baby seals, robotic dogs or even simple virtual assistants such as Alexa—present as having agency, sentience and an ‘inner life’ that they do not have.^6^ Does this make a difference to their appropriateness as trusted entities?

Coeckelbergh says, ‘Appearing-making, sometimes named ‘deception’, […] is part of ‘the social game’ and it does not undermine trust but supports it.’ (57) On one interpretation Coeckelbergh’s statement is true. It is ‘appearing-making’ that facilitates trust and in that sense it supports it—the agent-like appearance or behaviour paves the way for trust: it enables trust to develop. But on another interpretation the very fact that the behaviour that the trust is dependent on is deliberately fake, untrue and—even if for very good social reasons—designed to elicit false beliefs in humans, surely has the potential to undermine the very trust that it enables. For better or worse, the trust is directed towards a façade.

## Trust on the basis of misleading appearances

In ‘You Can Trust The Ladder, But You Shouldn’t’ (2019) [21] Jonathan Tallant argues that, although we can trust objects, we should not. Tallant considers a thought experiment in which an inanimate object appears to be the recipient of trust. The case that Tallant gives involves a child, Wiley, who is tricked by his sister into thinking that his blackboard is independently communicating with him—tricked into thinking that the blackboard has agency. Wiley is being misled and on discovering this he will show many of the attitudes that we associate with trust being betrayed. Tallant says:

‘[…] Wiley will have many of the “reactive attitudes” that are associated with a breach of trust […]. Wiley is being tricked into forming these attitudes towards the blackboard and seems to be engaging with the board as a moral agent; blaming it, resenting it, being disappointed by it, etc.’

Wiley trusts the blackboard. However, according to Tallant, Wiley should not trust the blackboard because, despite appearances, the blackboard is only an inanimate object. As a 5 year-old child, and given the circumstances that Tallant lays out in the paper, Wiley can be excused for believing that the blackboard has agency. But it does not, therefore trust is bestowed mistakenly. To clarify, for Tallant, the appearance of agency is enough to facilitate trust, but it is not enough to warrant trust, or make it appropriate. For that we need actual agency. So, according to Tallant, we have a case where trust can be given, but should not be. That seems like a reasonable position.

However, there is a relevant difference between the case of Wiley and our standard interactions with social robots. When we engage with social robots, even as we form emotional bonds with them and take attitudes of trust towards them, we remain aware on some level that they are objects without agency. To reap the benefits that the social robots offer, we have willingly bought in to the pretence. There is a kind of deception, but it is one that we seem happy to go along with, unlike in the case of Wiley and his blackboard.

Whilst accepting much of Coeckelbergh’s foundational analysis of why trust is possible towards social robots when it is not possible towards other objects, I propose that this position should be tempered by Tallant’s claim that the appearance of social interaction alone cannot legitimately facilitate trust. What we need, and what I propose, is a hybrid position that allows us to embrace our relationships with social robots while minimising the risks associated with trusting this particular kind of object.

Trusting social robots on the basis of appearances, whilst simultaneously being aware that those appearances are not manifestations of the relevant associated mental states, is very different in terms of risk from trusting humans on the basis of appearances. It is our social interactions with humans that have paved the way for our trust in social robots. Social robots are explicitly designed to mimic human-to-human interactions, yet they are very different beings from humans and in a way that is relevant to trust. As such, caution is required. However, if we are too cautious, we will not be in a position in to reap the full potential of advances in technology. It must continue to be possible for us to trust social robots, at least partly based on our social interactions with them.

The predicament we are in is this: social robots can bring a social good only if we trust them. But the very mechanism through which our trust is enabled has the potential to undermine our trust, as shown below.

- P1. The social interactions that we have with social robots facilitate our attitudes of trust towards them.
- P2.The interactions facilitate attitudes of trust in virtue of the robots being designed to mimic the kinds of social behaviours that humans display towards each other, behaviours that engender trust.
- P3.Human displays of the social behaviours that engender trust do so because they are (defeasibly) reliable evidence of a human’s cooperative attitudes.
- P4.Social robot displays of the social behaviours that engender trust are not evidence of their cooperative attitudes, but are entirely perfunctory.
- P5. We are trusting social robots on the basis of a faulty assumption—that the social behaviour social robots display is sufficient evidence of their cooperative attitude.

C-We are not warranted in trusting social robots.

Below, I will propose that P5 is false. We do not, in fact, assume that the behaviour of robots is evidence of a cooperative attitude. To frame my position I draw on the significant metaphysical differences between social robots and human or animal agents as described by the fictional dualism theory of social robots [1].

## Social robots, empathy and rights

Sweeney [1], engages with the question of whether social robots should be granted rights. As our ability to form strong emotional connections to social robots becomes more evident, the question of granting robot rights has gained prominence.^7^ There is a large body of evidence to suggest that, in the face of the anthropomorphic features that robots can display, we can care for robots, think of them as true companions or colleagues, consider their ‘feelings’ and their ‘mental state’ and even fall in love with them. Given how social robots can become woven into our lives, much as other people or pets might, we may consider granting them protective rights on that basis alone, regardless of their status as moral agents.

In addressing the question of rights [1] argues that we need a clearer understanding of what social robots are. It is proposed that social robots have a dual metaphysics: they are synthetic products or ‘tools’ with a fictional overlay. The fictional overlay is a character with mental states, beliefs, preferences, feelings and so forth. Importantly, the fictional overlay is entirely response-dependent—it is something that we individuals create in response to the anthropomorphic features that the social robot displays. The cuddly look and feel and the repeated friendly interaction of the PARO encourage me to project a character complete with mental states on to the robot which leads to my developing a relationship with it, despite my knowledge that PARO is a synthetic product or tool. This dual nature of social robots is a welcome feature. It is their dual nature that allows us to reap the social and health benefits that arise from our ability to become emotionally attached to them, whilst at the same time allowing us to fall short of granting legal or moral rights. For whilst we can temporarily give in to the emotional attachment, we can also remind ourselves that the inner life of agency that we project on to the object is a fiction that we have created. We are able to toggle between these two mindsets—between seeing the object as a fictional sentient being and seeing the object as a synthetic product.^8^ Here I propose that this same model can help us to understand why trust in social robots is possible, and also why we might want to set appropriate limits or safeguards on the trust that we bestow on social robots.

## Fictional dualism and trusting social robots

The fictional overlay part of the fictional dualism model of social robots coheres well with Coeckelbergh’s theory that it is the social—the shared experience—that is central when it comes to bestowing trust. However, according to the fictional dualism model, the robot must be understood as being more than just its social dimension—the robot is also a synthetic product that is in an important sense entirely distinct from its fictional, social dimension. During our interactions with social robots, we appear to display an ability to toggle between our attachment to the social robot that arises from our social engagement to it on the one hand, and our awareness that the robot is physically a synthetic product without full agency on the other. In this sense, our trust in social robots is importantly not like Wiley’s trust in the blackboard. Wiley believes that the backboard’s ‘behaviour’ is a result of its agency, but the human interlocutor with the social robot does not have this belief. When we engage with social robots on an emotional level we are, at the same time, aware that the robot’s human- or animal-like behaviour is a façade.^9^ In other words P5, above, is false. Although we are capable of going along with the fiction that our social robot is a trustworthy friend we are also aware on brief reflection that the behaviour is not representative of an attitude that the robot might take, because the robot does not have attitudes. Furthermore, we can indulge the friendship and camaraderie that comes from our interactions with the fictional overlay of our own particular PARO, let’s call her Snowy, while being aware that Snowy’s behaviour is not an indicator of the reliability of either that PARO itself, or PAROs in general. For Snowy’s behaviour could remain entirely the same while her hardware or software significantly alters in terms of reliability. This potentially places us at risk.

One might suggest that there is a sense in which human-to-human trust can also require an alertness to what is ‘on the inside’. When we decide to trust someone we do so on the basis of a judgement that is likely to be largely based on whether or not they present to us as trustworthy: do they behave in a friendly and cooperative way towards us, do they behave in a way that indicates that they understand what we are asking of them and the importance of the task? If so, we may well bestow trust on them. Of course, it is entirely possible that behind the façade of friendliness and care is an intention to hurt us, betray us and let us down. That is, with both humans and social robots, what is outwardly presented to the truster could be non-representative of what is going on ‘inside’—outward appearances can differ from what is going on behind the scenes. If this is the case, why should we let this feature of social robots make us particularly cautious about granting them trust? What precisely is the new risk?

There is a significant difference between our trust in social robots and trust in humans in this regard. The presentation of the social robot’s persona will *always* be a façade—it is never a representation of the social robot’s inner beliefs or intentions because those things do not exist. The anthropomorphic presentation is always a misrepresentation of the inner workings. With human-to-human interactions, while it is certainly possible for us to be misled in this way, it is not the norm. Importantly, if it were the norm, human-to-human trust would not be possible. Generally, people outwardly present in a way that resembles their inner beliefs and intentions and our practices of trusting depend on this. With social robots, there would be no stressful inner tension to be betrayed if it were programmed to behave with anthropomorphic behaviour that was designed to cultivate trust and friendship while simultaneously gathering and forwarding data about which products you might prefer to purchase. Social robots could easily be dangerous deceivers precisely because we are prone to accept them despite our knowledge that they are designed to present as something that they are not.

To summarise, it seems that the conditions for trusting social robots ought to be and arguably are different from the conditions for trusting humans. With humans, we assume that behaviour belies intention: an assumption that reinforces trust. With social robots we know that behaviour does not belie intention. We need something else to play that reinforcing role.

In section two we considered how objects such as ladders and blackboards cannot be recipients of trust but are instead appropriately judged in terms of reliability. Roughly speaking, an object is reliable if it functions in the way that it is reasonably expected to. I propose that the dualist nature of social robots allows us to understand our attitude of trust in social robots as being itself dependant on an assumption of product reliability, understood in a distinctive way. I propose that trusting social robots in a non-naïve way cannot be simply a reaction to the behaviour of the social robot, as Coeckelbergh proposes. Rather an implicit assumption of the reliability of the product must underpin our ability to engage with a social robot in way that engenders trust. It is this assumption of reliability that plays the role of the required link between the social behaviour of the robot and its inner workings. In trusting the robot we are assuming that the product is reliable for its advertised purpose of furthering our interests. Any evidence that works against this assumption of unreliability in the product will undermine our trust in the fictional overlay. To use our earlier example, any evidence of the unreliability of PARO, would destroy the attitude of trust we are inclined to take towards the fictional overlay, Snowy.

From the perspective of a consumer, PARO will continue to be assumed to be reliable as long as its product functions are, and if it functions in line with its advertised purpose. PARO can be deemed to be unreliable if it functions in additional ways that differ from its advertised purpose. For example, if PARO was secretly capable of sharing its clients’ personal data with other organisations, this would count towards our judgement of the reliability of the product as a companion for the consumer.

## Trusting social robots

Where does this leave us—can we trust the social robot? Yes, but only if our assumption of the reliability of the product remains intact. In that circumstance, we can form an attitude of trust towards the social robot on the basis of our social interactions with the fictional overlay. Recalling Snowy, I can trust Snowy because she makes me laugh, creates engaging dialogue and appears to care about my health and happiness. However, my trust in Snowy is vulnerable to the perceived reliability of that individual PARO and PAROs in general. If PARO is advertised as a cosy companion to help with loneliness, I am entitled to implicitly assume that it does not gather my personal data for other means. My trust in Snowy takes the reliability of the object PARO for granted. But if I read an article informing me that PAROs have been fitted with a device that records my private conversations and sells them to a marketing company I am likely to withdraw my trust of Snowy. My PARO is no longer a reliable object and this unreliability affects my ability to trust, despite our social interactions remaining the same.

None of this is to say that social robots are likely to be programmed to fulfil malign objectives—rather that, if they were, our social interactions with them would likely betray no sign of it. As such, when focusing on the social robot as an object of corporate design, one must be alert to indicators of unreliability alongside our willingness to form social attachments to the robot.

In sum, it is essential that we keep the dualist nature of social robots in mind, particularly because ‘what is inside’ the social robot can alter dramatically in ways that matter for trust, while what is presented—what is social—can stay the same. Yes, we are capable forming an attitude of trust towards social robots and, yes, we should continue to engage with the fiction to reap the full potential benefits of technological advancement. However, we should also bear in mind the nature of the entity that we are trusting and that the assumed reliability of the product is an essential basis of that trust. With social robots, far more so than with humans, all is not what it appears to be. As such it is important for us to regularly assess the reliability of the social robot as a technical product. An indication of non-reliability in the object—as an individual token, a product type or even in its production company—should undermine our attitude of trust towards the fictional overlay. That is, the behaviour of the social robot can remain the same, but if we discover something that throws the object’s reliability into doubt, the attitude of trust must be re-examined.

## Concluding remarks

We began by considering traditional philosophical theories of trust and how compatible they might be with our trusting social robots. The anthropomorphic elements that appear to be essential to many theories of trust would indicate that trust in objects is not possible, except perhaps in cases such as the one given by Tallant in which a child is purposefully deceived into thinking that a particular object has agency. However, there is evidence that we are capable of trusting social robots, despite our generally assuming that they do not have the levels of agency required for the kinds of cognitive attitudes that we have linked to trust. Coeckelbergh’s theory, that it is social relations that matters when it comes to trust, is helpful in showing a way forward—we trust social robots because they appear to us to have the kinds of agency compatible with trust. However, as the fictional dualism theory of social robots makes clear, social robots are not—in relevant and important ways—what they present to us to be. The presented characteristics are not indicative of attitudes in the way that human characteristics generally are. This opens us an important gap between what is outwardly presented and what might be going on ‘inside’. To bridge this gap we must recognise that the attitude of trust we take towards the social robot is underpinned by an implicit assumption regarding the reliability of the product. As such, when trusting social robots, we must remain alert to the possibility that the product could be or become unreliable, programmed with a purpose that goes beyond or is even at odds our personal health. Some awareness of the technological capabilities of the device and the purpose of system upgrades is advisable and relevant to our continued trust. It may be that the technological capabilities of the device are not currently easy to determine that the manufacturer is good at advertising the marketable features of the product, but less good at advertising the background features or abilities. Given our tendency to form attitudes of trust towards social robots and the risk that this unregulated tendency brings, this points to a need for greater responsibility to be taken by manufacturers. In particular, there is a need for transparency regarding the full range of the technological capabilities of the social robots that we bring into our home.

## Footnotes

1 See for example Hawley ([14], pp. 2029–2030) and Baier ([2], p. 235).

2 I will not give an analysis of trust here. I simply point out that, in the various ways that trust has been conceived, it is presented as being exclusionary of objects.

3 When faced with a proposed set of conditions for some concept, T, and a context, C, in which T appears to apply without meeting the conditions, we can either chose to adjust the concept or we can stick with the concept and argue that the appearance of T that we see in C is mistaken. In his (2020), Ryan goes for the latter, arguing that, as the traditional views of trust exclude trusting AI, we cannot consider AI as an appropriate recipient of trust.

4 Not everyone will be convinced that we can trust social robots, as opposed to simply relying on them but, as noted here, as humans are displaying trust behaviour and using trust language, there is certainly the possibility that an attitude of trust, and not just reliability, has been formed. Some theorists may want to insist that the attitude we have towards AI systems is not simply reliability but it also falls short of trust in some important way. For example, Taddeo [20] distinguishes e-trust (trust in digital environments) from trust and Grodzinsky et al. [10] use the notation TRUST to distinguish trust in digital environments from human-to-human, face-to-face trust. Others (for example, Bryson: 2010) think that trust in social robots is possible but dangerous and, as such, we should be careful not to humanise them.

5 Coeckelbergh discusses the work of Taddeo [20] and Floridi & Sanders [7] in which the notion of agency is reinterpreted in various ways to make the agency division between humans and robots blurry. I will not explore this option in this paper.

6 I am not taking a stand here on whether we can describe social robots as having agency in the technical sense. Floridi and Sanders [7] have argued, convincingly, that we can conceive of AI as meeting the requirements for moral agency. Here, I am more interested in a regular connection between behaviour and mental state.

7 See, for example, Sweeney [1], Gerdes [8], Darling [6], Bryson [3], Gunkel and Coeckelbergh [5], Gunkel [11] and Nyholm [16].

8 We perform a similar kind of toggling when we find ourselves devastated when our favourite character in a movie dies—to return to our ‘real’ lives without the devastation we remind ourselves that the character is not a real person.

9 The emotional response and the belief might appear to be at odds with one another, yet it is entirely coherent that I might feel devastated when my PARO ‘dies’—that I have lost something of great sentimental value, despite my simultaneous belief that my PARO is an inanimate object that can be replaced. As noted above, we can have a similar response to other kinds of fiction, feeling genuine sadness when a fictional character ‘dies’, despite our knowledge that, at least in the ordinary sense, they never existed.
